# Tofogliflozin, a sodium/glucose cotransporter 2 inhibitor, attenuates body weight gain and fat accumulation in diabetic and obese animal models

**DOI:** 10.1038/nutd.2014.20

**Published:** 2014-07-07

**Authors:** M Suzuki, M Takeda, A Kito, M Fukazawa, T Yata, M Yamamoto, T Nagata, T Fukuzawa, M Yamane, K Honda, Y Suzuki, Y Kawabe

**Affiliations:** 1 Research Division, Chugai Pharmaceutical Co. Ltd., Gotemba, Japan; 2 Chugai Research Institute for Medical Science Inc. Gotemba, Japan

## Abstract

**Objective:**

Tofogliflozin, a highly selective inhibitor of sodium/glucose cotransporter 2 (SGLT2), induces urinary glucose excretion (UGE), improves hyperglycemia and reduces body weight in patients with Type 2 diabetes (T2D). The mechanisms of tofogliflozin on body weight reduction were investigated in detail with obese and diabetic animal models.

**Methods:**

Diet-induced obese (DIO) rats and KKAy mice (a mouse model of diabetes with obesity) were fed diets containing tofogliflozin. Body weight, body composition, biochemical parameters and metabolic parameters were evaluated.

**Results:**

In DIO rats tofogliflozin was administered for 9 weeks, UGE was induced and body weight gain was attenuated. Body fat mass decreased without significant change in bone mass or lean body mass. Food consumption (FC) increased without change in energy expenditure, and deduced total calorie balance (deduced total calorie balance=FC−UGE−energy expenditure) decreased. Respiratory quotient (RQ) and plasma triglyceride (TG) level decreased, and plasma total ketone body (TKB) level increased. Moreover, plasma leptin level, adipocyte cell size and proportion of CD68-positive cells in mesenteric adipose tissue decreased. In KKAy mice, tofogliflozin was administered for 3 or 5 weeks, plasma glucose level and body weight gain decreased together with a reduction in liver weight and TG content without a reduction in body water content. Combination therapy with tofogliflozin and pioglitazone suppressed pioglitazone-induced body weight gain and reduced glycated hemoglobin level more effectively than monotherapy with either pioglitazone or tofogliflozin alone.

**Conclusion:**

Body weight reduction with tofogliflozin is mainly due to calorie loss with increased UGE. In addition, tofogliflozin also induces a metabolic shift from carbohydrate oxidation to fatty acid oxidation, which may lead to prevention of fat accumulation and inflammation in adipose tissue and liver. Tofogliflozin may have the potential to prevent obesity, hepatic steatosis and improve insulin resistance as well as hyperglycemia.

## Introduction

More than 340 million people worldwide have diabetes mellitus,^1^ ∼90% of whom have Type 2 diabetes (T2D). Epidemiological studies identify obesity as a major risk factor for T2D,^2, 3^ and intra-abdominal adiposity is profoundly associated with the pathogenesis of T2D via inflammation in adipose tissues, insulin resistance and impaired glucose regulation caused by fat accumulation.^4, 5^ Therefore, diet and exercise are regarded as an important strategy to prevent and delay progression of T2D.^6^ However, it is difficult to control body weight and plasma glucose solely by diet and exercise.^7, 8^

Furthermore, few antidiabetics have any antiobesity effect. Insulin analogues, insulin secretagogues and peroxisome proliferator-activated receptor γ agonists inevitably increase body weight,^9, 10^ and metformin^11^ and dipeptidyl peptidase 4 inhibitors^12^ do not obviously affect body weight. Although glucagon-like peptide 1 analogues can reduce body weight,^13^ they are used via subcutaneous self-injection and also have gastrointestinal side effects. Therefore, an orally available antidiabetic that can control both plasma glucose and body weight is required for T2D patients.

Sodium/glucose cotransporter 2 (SGLT2), which is expressed specifically in the proximal tubules of the kidney, has a dominant role in the renal glucose absorption.^14^ Recent clinical studies have indicated that oral administration of SGLT2 inhibitors induces urinary glucose excretion (UGE), improves hyperglycemia and reduces body weight of T2D patients.^15, 16, 17^ Tofogliflozin, a potent and highly selective SGLT2 inhibitor, induces UGE and improves hyperglycemia in rodents without risk of inducing hypoglycemia,^18, 19^ and in clinical studies, tofogliflozin improved hyperglycemia and reduced body weight.^20, 21^ However, the mechanism through which tofogliflozin reduces body weight is unclear. Here, we investigated the mechanism of body weight reduction with tofogliflozin by using diet-induced obese (DIO) rats as an obesity model and KKAy mice as an animal model of diabetes with obesity.

## Materials and methods

Lists of the reagents, animals, apparatuses and schedules for each experiment are summarized in Supplementary Table 1.

### Reagents and chemicals

Tofogliflozin was synthesized^22^ in our laboratories at Chugai Pharmaceutical Co, Gotemba, Japan. Pioglitazone hydrochloride (pioglitazone) was purchased from Sequoia Research Products Ltd (Pangbourne, UK). We prepared a powdered high-fat diet (HFD, 60% kcal fat, D-12492 (Research Diets Inc, New Brunswick, NJ, USA)) containing 0.05% tofogliflozin (HFD/TOFO), rodent diet (CE-2 (Clea Japan, Tokyo, Japan)) containing 0.015 or 0.0015% tofogliflozin (CE-2/TOFO), CE-2 containing 0.02% pioglitazone (CE-2/PIO) and CE-2 containing 0.02% pioglitazone plus 0.0015% tofogliflozin (CE-2/PIO+TOFO).

### Animals

Male Wistar rats (Jcl:Wistar) and KKAy mice (KKAy/TaJcl) purchased from Clea Japan were housed under a 12-h/12-h light/dark cycle (lights on 0700 –1900 hours) with controlled room temperature (20–26 °C) and humidity (35–75%), and allowed free access to food (CE-2) and water. All animal care and experiments followed the guidelines for the care and use of laboratory animals at the Chugai Pharmaceutical Co.

### Effect of tofogliflozin in DIO rats

#### General methods

Twenty-one male Wistar rats (8-week-old), randomly allocated into three groups matched for plasma glucose and body weight, were housed individually with free access to food and water. The normal diet (ND) and HFD groups were fed for 13 weeks a powdered ND (10% kcal fat, D-12450B (Research Diets Inc.)) and powdered HFD, respectively. The TOFO group was fed HFD for 4 weeks and HFD/TOFO for an additional 9 weeks. Week 1 was defined as when feeding with HFD/TOFO started. Body weight and food consumption (FC) were measured periodically. Hematocrit was measured in blood collected via the jugular vein by Hematocrit Capillary (VC-H075P (Terumo Co, Tokyo, Japan)) at Weeks 5 and 9. Rectal temperature was measured with a microprobe thermometer (BAT-12 (Physitemp Instruments Inc, Clifton, NJ, USA)). Plasma tofogliflozin concentration was measured in blood collected via the jugular vein or the tail vein as described previously.^18^ To determine the following biochemical parameters, blood was sampled via the jugular vein of non-fasted rats then centrifuged to obtain plasma samples. Plasma insulin and leptin concentrations were measured with ELISA kits (Morinaga Institute of Biological Science Inc, Yokohama, Japan). Plasma glucose, triglyceride (TG), total cholesterol (TC) and total ketone bodies (TKBs) were measured with an automated analyzer (TBA-120FR (Toshiba Medical Systems Co, Tochigi, Japan)). At the end of the study (Week 10), rats were killed by exsanguination under anesthesia and adipose tissues (mesenteric, epididymal, inguinal, and retroperitoneal adipose tissues) and skeletal muscle (soleus) were isolated. The tissues were weighed and stored at −80 °C or in 10% formalin solution until use.

#### Body composition by microcomputed tomography (microCT)

Rats were anesthetized with isoflurane, and body composition evaluated with a microCT scanner (eXplore Locus (General Electronic Company, Tokyo, Japan)) and the image analysis software (MicroView (General Electronic Company)).^23^ The CT value of olive oil (Wako Pure Chemical Industries Ltd, Osaka, Japan) was used as standard density for adipose tissue. The CT value of a standard phantom with calibration cells containing calcium hydroxyapatite (Kyoto Kagaku, Kyoto, Japan) at a concentration equivalent to 400 mg cm^−3^ was used as standard density for bone. A voxel with a CT value higher than that of the standard phantom was defined as bone. A voxel with CT value between that of the standard phantom and olive oil was defined as the lean body mass.

#### Quantitative RT-PCR for CD14 and CD68 mRNA

Total RNA was isolated from the frozen mesenteric adipose tissue with TRIzol reagent (Life Technologies Co., Carlsbad, CA, USA) and an RNeasy 96 kit (Qiagen, Venlo, the Netherlands). The relative amounts of CD14 and CD68 mRNA in the total RNA were calculated with 18S rRNA as an internal control using a real-time RT-PCR quantitative system (7900HT Fast Real Time PCR system (Applied Biosystems, Foster City, CA, USA)). The RT-PCR reaction was performed with a QuantiTect Probe RT-PCR kit (Qiagen) and probes (TaqMan Gene Expression Assays for rat, CD14: Rn00572656_g1, CD68: Rn01495634_g1, 18S rRNA: Hs99999901_s1 (Applied Biosystems)).

#### Cell size of adipocytes

Mesenteric adipose tissue was fixed in phosphate-buffered 10% formaldehyde (pH 7.2) and embedded in a paraffin block. The paraffin block was sliced 7-μm thick and stained with hematoxylin–eosin by Sapporo General Pathology Laboratory Co. (Sapporo, Japan). In two microscopic images selected at random per section, the cell size (surface area) of all adipocytes (∼150 cells) in the images was measured with the image analysis software (NIS-Elements D2.20 SP1 (Nikon Co., Tokyo, Japan)).

#### Immunohistochemical staining

The block of paraffin-embedded mesenteric adipose tissue was sliced 4-μm thick and stained with anti-rat CD68 (ED-1) monoclonal antibody (monocyte/macrophage marker; No. MCA341R (AbD Serotec, Kidlington, UK)) as a primary antibody, anti-mouse/rabbit IgG-HRP (LSAB2 System-HRP, No. K0609 (Dako Denmark A/S, Glostrup, Denmark)) as a secondary antibody, and a chromogenic substrate for HRP (DAB+, Liquid, K3468 (Dako Denmark A/S)) by Sapporo General Pathology Laboratory Co. In five microscopic images selected at random per section, the CD68-positive area was calculated as a percentage of total area with an eSlide Capture Device (Aperio ScanScope CS (Aperio Technologies, Vista, CA, USA)) and image analysis software (Image scope (Aperio Technologies)).

#### UGE

Rats were housed individually in metabolic cages for 24 h and urine samples were collected and urine volume measured. Urinary glucose concentration was measured by the hexokinase method (Autosera S GLU (Sekisui Medical Co, Tokyo, Japan)).

#### Indirect calorimetry and locomotor activity

At week 9, rats were housed in metabolic chambers (AC-001R (Muromachi Kikai Co, Tokyo, Japan)) with free access to food and water for 24 h. Air was sampled every 5 min and consumed oxygen (VO_2_ (ml min^−1^)) and produced carbon dioxide (VCO_2_ (ml min^−1^)) were measured by an O_2_/CO_2_ metabolic measuring system (MM208 (Muromachi Kikai Co.)). From these we calculated respiratory quotient (RQ=VCO_2_/VO_2_) and energy expenditure (energy expenditure=1.07 × RQ × VO_2_+3.98 × VO_2_ (cal min^−1^)). At the same time, locomotor activity was monitored every minute using a Supermex multichannel activity-counting system (Animex Auto MK-110 (Muromachi Kikai Co)).

### Effect of tofogliflozin in KKAy mice

#### Experiment 1: effect on plasma glucose, body weight and liver TG content

Twenty-four male KKAy mice (8-week-old), randomly allocated into two groups matched for plasma glucose and body weight, were fed CE-2 or CE-2/TOFO (0.015%) for 5 weeks. The mice were caged individually with free access to food and water. The day on which the mice began to be fed CE-2/TOFO is defined as Day 1. Body weight and FC were measured periodically. Blood was sampled via the tail vein, and plasma glucose level was measured using blood glucose monitoring system (ACCU-Check Aviva (Roche Diagnostics, Tokyo, Japan)). Glycated hemoglobin (Hb) level was measured with an automated analyzer (Auto Wako HbA1c (Wako Pure Chemical Industries Ltd)). Plasma TG and TC levels were measured with the automated analyzer. Plasma insulin and adiponectin levels were measured with ELISA kits (insulin: Morinaga Institute of Biological Science Inc; adiponectin: Otsuka Pharmaceutical Co, Tokyo, Japan). Between Day 29 and Day 30, mice were housed individually in metabolic cages for 24 h, and the urine volume and urinary glucose concentration were measured. At the end of the study (Day 35), mice were killed by exsanguination under anesthesia, and then their livers were isolated, weighed and stored at −80 °C until use. The liver was homogenized in methanol and a twofold volume of chloroform was added and mixed, and then centrifuged at 1200 *g* for 10 min. The supernatant was collected, dried and dissolved in isopropyl alcohol containing 10% Triton X-100. TG concentration in the solution was measured with a Triglyceride E-test kit (Wako Pure Chemical Industries Ltd)), and liver TG content (mg per g tissue) was calculated.

#### Experiment 2: effect on body water content

Thirty-two male KKAy mice (8-week-old) were randomly allocated into two groups (CE-2 and CE-2/TOFO (0.015%)) matched for plasma glucose and body weight. On Day 3 or Day 20, body weight was measured and the mice were killed by cervical dislocation. The dead body was dried at 65 °C for 17 days and dry body weight was measured. Body water content of the mouse was calculated as follows:


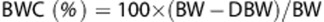


where BWC is body water content, BW is body weight and DBW is dry body weight.

#### Experiment 3: effect on body weight gain induced by pioglitazone

Twenty-four male KKAy mice (8-week-old), randomly allocated into four groups matched for plasma glucose and body weight, were fed CE-2, CE-2/PIO, CE-2/TOFO (0.0015%) or CE-2/PIO+TOFO for 4 weeks. Body weight, FC, plasma glucose and glycated Hb levels were measured by the same methods as in Experiment 1.

### Statistical analysis

Data are presented as mean±s.e.m. Statistical analysis was performed with SAS System for Windows, Release 8.02 (SAS Institute Japan, Tokyo, Japan). Statistical significance was determined by Student's *t*-test, Dunnett's multiple comparison test or analysis of covariance. Multiregression analysis was performed with Microsoft Excel 2007 SP3 (Microsoft Corporation, Redmond, WA, USA).

## Results

### Body weight gain and fat accumulation in DIO rats

**Figure 1:**
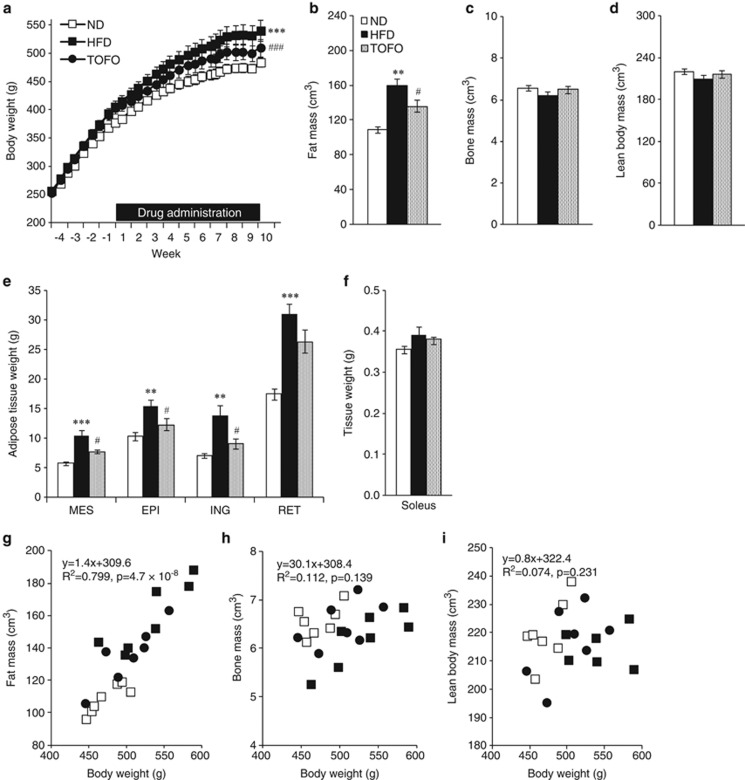
Effects of long-term administration of tofogliflozin on body weight and body composition of DIO rats. (**a**) Time course of body weight of rats fed ND, HFD or HFD/TOFO for 9 weeks. (**b**–**d**) Fat (**b**), bone (**c**) and lean body mass (**d**) calculated from microCT scanner images from the level of the fifth cervical vertebra to the second caudal vertebra at Week 8. (**e**–**f**) The weight of adipose tissues (**e**) and soleus (**f**) measured for mesenteric (MES), epididymal (EPI), inguinal (ING) and retroperitoneal (RET) adipose tissues and the soleus isolated at Week 10. (**g**–**i**) Body weight plotted against fat mass (**g**), bone mass (**h**) or lean body mass (**i**). Spearman correlation coefficient between body weight and each parameter is shown in the graph. Data (**a–f**) are expressed as mean±s.e.m. (*n*=7). **P<0.01 or ***P<0.001 versus ND group, and ^#^*P*<0.05 or ^###^*P*<0.001 versus HFD by analysis of covariance (ANCOVA; **a**) or Student's *t*-test (**b**–**f**).

Body weight increased more rapidly (Figure 1a) in HFD group (134.4±9.6 g per 9 weeks) than in the ND group (106.6±2.8 g per 9 weeks), and the final body weight was greater in the HFD group (540±18.5 g) than in the ND group (483.9±7.7 g). HFD feeding increased total fat mass by ∼46% (Figure 1b). On the other hand, there was no change in bone mass (Figure 1c) or lean body mass (Figure 1d) between the groups. HFD feeding increased weights of all adipose tissues (Figure 1e) without increasing soleus weight (Figure 1f).

In rats fed HFD/TOFO (TOFO group), body weight increased more slowly than in the HFD group, and the final body weight was ∼30 g lower in the TOFO group than in the HFD group (Figure 1a), which was almost equivalent to the HFD-induced body weight gain versus the ND group (∼28 g) mentioned above.

The enlargement of total fat mass decreased by 14.6% in the TOFO group compared with the HFD group (Figure 1b). In contrast, there was no change in bone mass or lean body mass between HFD and TOFO groups (Figures 1c and d). Tofogliflozin administration prevented HFD-induced enlargement of adipose tissues (Figure 1e) without reducing soleus weight (Figure 1f).

Body weight correlated positively with fat mass (*R*^2^=0.799, *P*=4.7 × 10^−8^; Figure 1g); however, there was no correlation with bone mass (Figure 1h) or lean body mass (Figure 1i). Multiple regression analysis performed with body weight gain between Week 1 and Week 8 as a dependent variable, and fat, bone and lean body mass as independent variables (Supplementary Table 2) found that body weight gain was mainly accounted for by increased fat mass (*P*=7.01 × 10^−6^).

### Cell size of adipocytes and monocyte/macrophage infiltration into mesenteric adipose tissue in DIO rats

**Figure 2:**
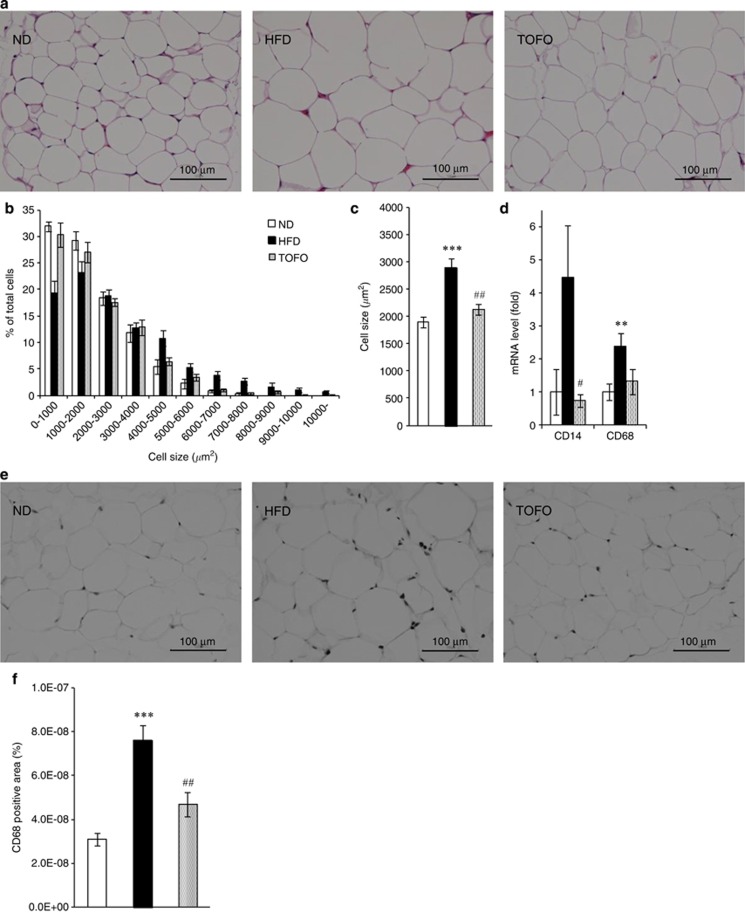
Effects of long-term administration of tofogliflozin on the cell size of adipocytes and infiltration of inflammatory cells into mesenteric adipose tissue in DIO rats. (**a**) Representative images of hematoxylin–eosin-stained sections of mesenteric adipose tissue isolated at Week 10. (**b**, **c**) Distribution (**b**) and the mean value (**c**) of cell size of adipocytes. (**d**) mRNA level of CD14 and CD68 in mesenteric adipose tissue relative to level in the ND group. (**e**) Representative images of sections stained with anti-rat CD68 (monocyte/macrophage marker) monoclonal antibody. (**f**) Percentage of CD68-positive area in microscopic images. Data (**b**–**d** and **f**) are expressed as mean±s.e.m. (*n*=7). **P<0.01 or ***P<0.001 versus ND group, and ^#^*P*<0.05 or ^##^*P*<0.01 versus HFD by Student's *t*-test.

To evaluate the effects of tofogliflozin on the development of adipose tissue *in vivo*, the cell size of mesenteric adipose tissue was compared among treatment groups (Figure 2a). Cell size distribution was shifted to the larger direction in HFD group compared with the ND group (Figure 2b). The mean cell size was larger in the HFD group than in the ND group (Figure 2c). The mRNA levels of the monocyte/macrophage markers CD14 and CD68 in mesenteric adipose tissue in the HFD group increased to 4.5 times and 2.4 times (respectively) the levels in ND group (Figure 2d). Immunohistochemical staining indicated that the area positive for CD68 (ED-1) was larger in the HFD group than in the ND group (Figures 2e and f).

In the TOFO group, the shift in adipose cell size distribution to the larger direction induced by HFD feeding was suppressed (Figure 2b), resulting in a significant reduction in the mean adipose cell size in the TOFO group as compared with the HFD group (Figure 2c). In addition, tofogliflozin administration decreased CD14 and CD68 mRNA levels as compared with the HFD group (Figure 2d; CD14: *P*=0.033, CD68: *P*=0.074), and reduced the CD68 (ED-1)-positive area in mesenteric adipose tissue (Figures 2e and f).

### Total calorie balance in DIO rats

**Figure 3:**
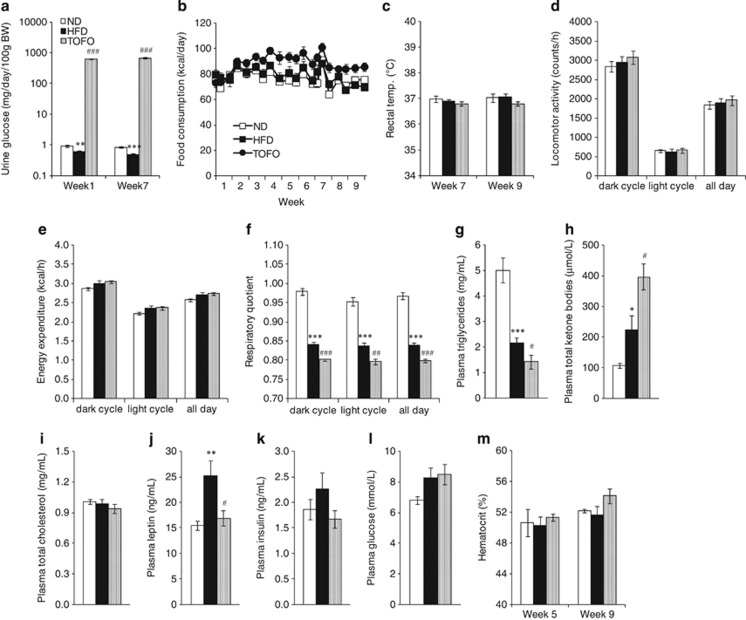
Effects of long-term administration of tofogliflozin on energy balance and metabolism of DIO rats. (**a**) Urinary glucose excretion at Week 1 and Week 7. (**b**) Food consumption. (**c**) Rectal temperature at Week 7 and Week 9. (**d**–**f**) Locomotor activity (**d**), energy expenditure (**e**) and respiratory quotient (**f**) measured with metabolic chamber at Week 9. (**g**–**l**) Plasma triglyceride (**g**), total ketone bodies (**h**), total cholesterol (**i**), leptin (**j**), insulin (**k**) and glucose (**l**) levels determined at Week 9. (**m**) Hematocrit level evaluated at Week 5 and Week 9. Data are expressed as mean±s.e.m. (*n*=7). Statistical comparisons (**a**, **c**–**m**) were performed by Student's *t*-test test: *P<0.05, **P<0.01, ***P<0.01 versus ND group, and ^#^*P*<0.05, ^##^*P*<0.01, ^###^*P*<0.001 versus HFD.

The plasma tofogliflozin concentrations in the TOFO group were 658±40 ng ml^−1^ at Week 1, 558±33 ng ml^−1^ at Week 5 and 509±50 ng ml^−1^ at Week 9. Tofogliflozin administration induced UGE at both Week 1 and Week 7 by 628±28 and 674±32 mg per day per 100 g BW (Figure 3a), which are estimated as calorie losses of 9.7±0.6 and 12.6±0.6 kcal per day, respectively. At the same time, calorie intake via FC from Week 1 to Week 9 in TOFO group (5718±187 kcal) was greater by ∼10% than that in the HFD group (5049±214 kcal, Figure 3b). On the other hand, no changes in rectal temperature (Figure 3c), locomotor activity (Figure 3d) or energy expenditure (Figure 3e) were observed between HFD and TOFO groups.

Thus, tofogliflozin administration increased both calorie loss by promoting UGE and calorie intake by increasing FC without altering energy utilization (Table 1). We assumed that the calorie loss (∼13 kcal per day) was greater than the increase in calorie intake (∼10 kcal per day), and the deduced total calorie balance decreased by ∼3 kcal per day in the TOFO group as compared with the HFD group.

### Metabolic balance between carbohydrate oxidation and fatty acid oxidation, and other biochemical parameters in DIO rats

RQ values of the ND group were stable at ∼0.95 in both light and dark periods. In contrast, RQ values of the HFD group were decreased to ∼0.85 in both light and dark periods (Figure 3f), indicating an increased rate of fatty acid oxidation with HFD. Plasma TG level was lower in the HFD group than in the ND group (Figure 3g), whereas TKB level was higher in the HFD group than in the ND group (Figure 3h). There was no difference in plasma TC level among the groups (Figure 3i). Plasma leptin level was greater in the HFD group than in the ND group (Figure 3j). Plasma insulin level in HFD and ND groups was 2.3±0.31 and 1.9±0.2 ng ml^−1^, respectively (ND versus HFD: *P*=0.295, Figure 3k). There were no differences between HFD and ND groups in plasma glucose levels at Week 9 (Figure 3l) or hematocrit levels at Week 5 and Week 9 (Figure 3m).

RQ value was further decreased by tofogliflozin to ∼0.80 (Figure 3f). Tofogliflozin also reduced plasma TG level (Figure 3g) but increased TKB level (Figure 3h) as compared with the HFD group. Plasma TC level was not changed by tofogliflozin (Figure 3i). In the TOFO group, the HFD-induced increase in plasma leptin was prevented (Figure 3j) and a tendency to decreasing plasma insulin (1.7±0.2 ng ml^−1^, Figure 3k) was observed. There were no differences between HFD and TOFO groups in plasma glucose (Figure 3l), which may be due to the compensatory increase in endogenous glucose production in the TOFO group responding to the increased UGE,^19^ or hematocrit levels (Figure 3m).

### Body weight gain and hyperglycemia in KKAy mice

#### Experiment 1: body weight, plasma glucose and biochemical parameters in long-term treatment

**Figure 4:**
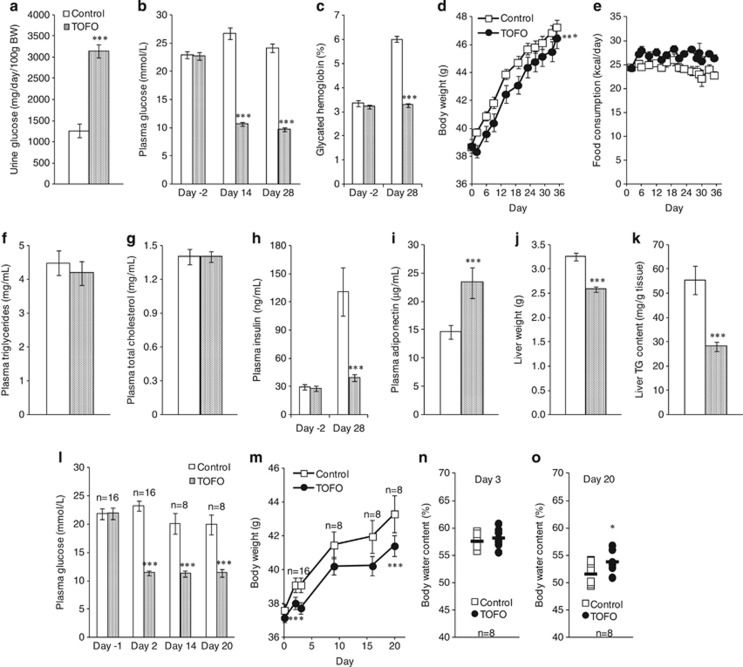
Effects of long-term administration of tofogliflozin on the plasma glucose and body weight of KKAy mice. Experiment 1: (**a**) urinary glucose excretion for 24 h from Day 29 to Day 30. (**b**) Plasma glucose levels on Day −2, Day 14 and Day 28. (**c**) Glycated hemoglobin (%) on Day −2 and Day 28. (**d**) Time course of body weight. (**e**) Food consumption between Day 0 and Day 35. (**f**–**i**) Plasma triglyceride (**f**), total cholesterol (**g**), insulin (**h**) and adiponectin (**i**) determined on Day 28. (**j**, **k**) The weight (**j**) and triglyceride (TG) content (**i**) of the liver sampled on Day 35. Data are expressed as mean±s.e.m. (*n*=12). Experiment 2: (**l**) plasma glucose levels on Day −1, Day 2, Day 14 and Day 20. (**m**) Time course of body weight. (**n**, **o**) Body water content of mice on Day 3 or Day 20. Data are expressed as mean±s.e.m. (*n*=8 or 16). Statistical comparisons were performed by Student's *t*-test (**a**–**c**, **f**–**l**, **n** and **o**) or by ANCOVA (**e** and **m**). *P<0.05 and ***P<0.001 versus control group.

Plasma tofogliflozin concentrations in the TOFO group were 546±69 ng ml^−1^ on Day 1 and 402±37 ng ml^−1^ on Day 28. Although an apparent UGE was observed in control group of KKAy mice on Day 29, tofogliflozin administration increased UGE by ∼2.5 times that of the control group (Figure 4a). In control group, plasma glucose level was maintained above 22 mmol l^−1^ (Figure 4b), and glycated Hb level increased from Day −2 to Day 28 by ∼1.8-fold (Figure 4c). Tofogliflozin administration reduced plasma glucose to below 11 mmol l^−1^, and prevented the increase in glycated Hb. Body weight of control group increased by 8.6±0.5 g over 35 days (Figure 4d). Although FC increased ∼10% in the TOFO group (948±18 kcal per 35 days) compared with control group (858±23 kcal per 35 days, Figure 4e), tofogliflozin administration attenuated body weight gain (Figure 4d).

There were no differences in plasma TG and TC level between the two groups on Day 28 (Figures 4f and g). Although plasma insulin level in control group increased from Day −2 to Day 28 by about 4.5-fold, tofogliflozin administration prevented this hyperinsulinemia (Figure 4h). Plasma adiponectin level was greater in the TOFO group than in control group (Figure 4i). The hypertrophy of the liver with steatosis observed in control group was suppressed by tofogliflozin administration (Figures 4j and k).

#### Experiment 2: body water content with 3- or 20-day treatment

Tofogliflozin administration again reduced plasma glucose to below 12 mmol l^−1^ throughout the course of the experiment (Figure 4l). Tofogliflozin administration also decreased body weight gain, resulting in a significant difference in body weight between control and TOFO groups (Figure 4m) on Day 3 and Day 20. However, there was no significant reduction in body water content on Day 3 or Day 20 in the TOFO group (Figures 4n and o) as compared with control group.

#### Experiment 3: effect on body weight gain induced by 28-day pioglitazone treatment

**Figure 5:**
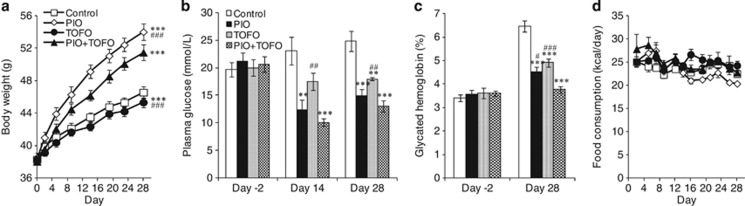
Effects of long-term administration of tofogliflozin on pioglitazone-induced body weight gain of KKAy mice. (**a**) Time course of body weight. (**b**) Plasma glucose levels on Day −2, Day 14 and Day 28. (**c**) Glycated hemoglobin (%) on Day −2 and Day 28. (**d**) Food consumption between Day 0 and Day 28. Data are expressed as mean±s.e.m. (*n*=6). Statistical comparisons were performed by Dunnett's multiple comparison test (**b**, **c**) or by ANCOVA (**a**). **P<0.01, ***P<0.001 versus control group; ^#^*P*<0.05, ^##^*P*<0.01, ^###^*P*<0.001 versus PIO+TOFO group.

Similar to Experiment 1, the body weight of control group increased by 8.2±0.2 g over 28 days (Figure 5a). Tofogliflozin administration again attenuated body weight gain (7.0±0.4 g per 28 days) as compared with control group. Pioglitazone administration increased the body weight more rapidly by 15.5±0.6 g over 28 days as compared with control group. The combined administration of pioglitazone and tofogliflozin reduced the body weight gained with pioglitazone (Figure 5a).

Pioglitazone administration reduced plasma glucose (Figure 5b) and glycated Hb levels (Figure 5c) on Day 28 as compared with control group. Tofogliflozin administration also reduced plasma glucose and glycated Hb levels, but the plasma glucose lowering effects of tofogliflozin (0.0015%) on Day 14 and Day 28 were slightly smaller than those of pioglitazone (0.02%). Combined administration of pioglitazone and tofogliflozin additively reduced plasma glucose and glycated Hb levels, resulting in a significant reduction in plasma glucose levels in the PIO+TOFO group versus the TOFO group on Day 14 and Day 28 (Figure 5b), and in glycated Hb levels in the PIO+TOFO group versus PIO and TOFO groups on Day 28 (Figure 5c). In this experiment, FC was not changed in the PIO group (652±12 kcal per 28 days), TOFO group (700±14 kcal per 28 days) or PIO+TOFO group (705±20 kcal per 28 days) as compared with control group (664±18 kcal per 28 days), and FC was not changed in the PIO+TOFO group as compared with PIO or TOFO group (Figure 5d).

## Discussion

In this study, excess body weight gain was attenuated with tofogliflozin in both obese DIO rats and diabetic KKAy mice. In DIO rats treated with tofogliflozin, enlargement of fat mass was suppressed without reduction of lean body mass or bone mass. In addition, suppression of body weight with tofogliflozin was accompanied with amelioration of hepatic steatosis in KKAy mice without a change in body water content. These results were consistent with other preclinical studies^24, 25, 26^ and a clinical study^27^ with SGLT2 inhibitors.

Although tofogliflozin reduced body weight in clinical studies,^20, 21^ its mechanism was unclear. In db/db mice, an animal model of T2D, although tofogliflozin treatment improved hyperglycemia, the body weight was paradoxically increased as compared with the untreated control group,^28^ which was possibly due to preserved insulin secretion in the tofogliflozin group, similarly to other SGLT2 inhibitors.^29^ Thus, it is difficult to evaluate the mechanisms of body weight reduction with SGLT2 inhibitors when using diabetic animal models with dysfunctional insulin secretion.

Therefore, we used DIO rats and KKAy mice in which insulin secretion is maintained and successfully found that tofogliflozin ameliorated of body weight gain. Moreover, body weight gain induced by pioglitazone was suppressed in KKAy mice treated with tofogliflozin.

In DIO rats treated with tofogliflozin, calorie loss by UGE exceeded the increased calorie intake, and deduced total calorie balance was lower than in the HFD group. Accumulated total calorie loss calculated by deduced total calorie balance was ∼190 kcal per head between Week 1 and Week 8, which was equivalent to the decreased fat mass (23.2 cm^3^) with tofogliflozin. These results suggest that tofogliflozin induces total calorie loss by increasing UGE and attenuates obesity predominantly by reducing fat accumulation. A similar mechanism for body weight reduction was proposed in the preclinical study with another SGLT2 inhibitor.^25^

In this study, body weight reduction was not associated with increased hematocrit in DIO rats or decreased body water content in KKAy mice, suggesting that body weight reduction was not caused by dehydration in these animal models.

In both DIO rats and KKAy mice treated with tofogliflozin, FC increased. Tofogliflozin prevented fat accumulation and reduced plasma leptin level in DIO rats, and improved hyperglycemia and hyperinsulinemia in KKAy mice. Reduction of insulin and leptin—hormones regulating appetite and FC^30^—might increase FC; however, tofogliflozin increased FC even in db/db mice^28^ and KKAy mice—animal models with low sensitivity to insulin and leptin—implying that other mechanisms might contribute to the regulation of their FC. Further analysis will be needed to clarify the mechanisms underlying the increased FC to compensate for the calorie loss and plasma glucose reduction.

One of the possible compensatory mechanisms for reduced plasma glucose level is lipolysis in adipose tissues, which generates glycerol and free fatty acids. Released glycerol is used for the endogenous glucose production in the liver, and the free fatty acids are used for ATP production with β-oxidation, leading to the increased production of ketone bodies,^31^ which are important energy substrates for the skeletal muscle, heart and brain.

Compared with the HFD group, in DIO rats treated with tofogliflozin, RQ decreased by ∼0.04, plasma TKB increased to ∼180 μmol l^−1^, and volume and weight of adipose tissues were reduced. These results suggest that the above-mentioned compensatory metabolic changes were induced by the treatment with tofogliflozin. The same phenomena were observed in other preclinical studies with SGLT2 inhibitors.^24, 25, 26^ In particular, in DIO mice treated with tofogliflozin increased fatty acid oxidation was observed with the upregulated expression of liver genes linked to stimulation of fatty acid oxidation such as proliferator-activated receptor α or carnitine palmitoyltransferase 1 in the liver.^24^ A slight increase in plasma TKB was also reported in T2D patients treated with SGLT2 inhibitors.^32^ These results indicate that treatment with SGLT2 inhibitors induces a metabolic shift from carbohydrate oxidation to fatty acid oxidation in order to compensate for glucose loss, thereby promoting fat utilization.

Obesity-induced fat accumulation in adipose tissues and liver, inflammation in adipose tissues, increased fatty acid synthesis in the liver and increased TG level in the plasma are associated with insulin resistance.^5, 33^ Amelioration of these pathological conditions observed in DIO rats and KKAy mice treated with tofogliflozin suggests that long-term treatment with tofogliflozin may improve insulin resistance in T2D patients.

Extreme liver steatosis is also associated with insulin resistance, inflammation and fibrosis in the liver, or nonalcoholic steatohepatitis.^34^ In KKAy mice, tofogliflozin improved hepatic steatosis, which is consistent with the effects seen in DIO mice treated with tofogliflozin^24^ and in nonalcoholic steatohepatitis animal models treated with another SGLT2 inhibitor.^35^ These results suggest that long-term administration of SGLT2 inhibitors may also prevent the progression of nonalcoholic steatohepatitis in T2D patients with obesity.

Pioglitazone, a proliferator-activated receptor γ agonist, improves insulin resistance and hyperglycemia in T2D patients but induces body weight gain^10^ with an increase in fat mass and fluid retention. Although pioglitazone induced body weight gain in KKAy mice, this effect was suppressed by tofogliflozin. In addition, combination therapy with tofogliflozin and pioglitazone improved hyperglycemia more effectively than did monotherapy with either tofogliflozin or pioglitazone alone. This combination therapy might be an attractive choice for T2D treatment.

The present study indicated that tofogliflozin promotes UGE and calorie loss and prevents body weight gain predominantly by ameliorating fat accumulation in the adipose tissue and liver. Tofogliflozin may have the potential to prevent or improve not only hyperglycemia but also insulin sensitivity by suppressing TG accumulation and inflammation in the adipose tissue and liver. Further studies are required to evaluate the effect of long-term treatment with tofogliflozin on insulin resistance in T2D patients.

## Figures and Tables

**Table 1:** Effect of long-term administration of tofogliflozin on deduced total calorie balance in DIO rats

*Group*	*Estimated energy balance (kcal per day)*			
	*Food intake*	*Energy expenditure*	*Urinary glucose excretion*	*Total*
ND	77.7±1.7	−61.0±0.8	0.0±0.0	16.6±1.7
HFD	81.0±3.5	−64.2±1.6	0.0±0.0	16.8±3.0
TOFO	90.8±3.1	−64.8±0.9	−12.6±0.6	13.4±2.3

Abbreviations: DIO, Diet-induced obese; HFD, high-fat diet; ND, normal diet; TOFO, tofogliflozin.

## References

[bib1] World Health Organization (2012) Fact sheet No. 312 “Diabetes”.

[bib2] Lipton RB, Liao Y, Cao G, Cooper RS, McGee D. Determinants of incident non-insulin-dependent diabetes mellitus among blacks and whites in a national sample. The NHANES I Epidemiologic Follow-up Study. Am J Epidemiol 1993; 138: 826–839. 8237971 10.1093/oxfordjournals.aje.a116786

[bib3] Chan JM, Rimm EB, Colditz GA, Stampfer MJ, Willett WC. Obesity, fat distribution, and weight gain as risk factors for clinical diabetes in men. Diabetes Care 1994; 17: 961–969. 7988316 10.2337/diacare.17.9.961

[bib4] James WP. What are the health risks? The medical consequences of obesity and its health risks. Exp Clin Endocrinol Diabetes 1998; 106(Suppl 2): 1–6. 10.1055/s-0029-1212028 9792473

[bib5] Olefsky JM, Glass CK. Macrophages, inflammation, and insulin resistance. Annu Rev Physiol 2010; 72: 219–246. 20148674 10.1146/annurev-physiol-021909-135846

[bib6] Israili ZH. Advances in the treatment of type 2 diabetes mellitus. Am J Ther 2011; 18: 117–152. 19834322 10.1097/MJT.0b013e3181afbf51

[bib7] American Diabetes Association. Standards of medical care in diabetes—2013. Diabetes Care 2013; 36(Suppl 1): S11–S66. 23264422 10.2337/dc13-S011 PMC3537269

[bib8] Esposito K, Maiorino MI, Ciotola M, Di Palo C, Scognamiglio P, Gicchino M et al. Effects of a Mediterranean-style diet on the need for antihyperglycemic drug therapy in patients with newly diagnosed type 2 diabetes: a randomized trial. Ann Intern Med 2009; 151: 306–314. 19721018 10.7326/0003-4819-151-5-200909010-00004

[bib9] Erlich DR, Slawson DC, Shaughnessy A. Diabetes update: new drugs to manage type 2 diabetes. FP Essent 2013; 408: 20–24. 23690375

[bib10] Hermansen K, Mortensen LS. Bodyweight changes associated with antihyperglycaemic agents in type 2 diabetes mellitus. Drug Saf 2007; 30: 1127–1142. 18035865 10.2165/00002018-200730120-00005

[bib11] Intensive blood-glucose control with sulphonylureas or insulin compared with conventional treatment and risk of complications in patients with type 2 diabetes (UKPDS 33). UK Prospective Diabetes Study (UKPDS) Group. Lancet 1998; 352: 837–853. 9742976

[bib12] Karagiannis T, Paschos P, Paletas K, Matthews DR, Tsapas A. Dipeptidyl peptidase-4 inhibitors for treatment of type 2 diabetes mellitus in the clinical setting: systematic review and meta-analysis. Br Med J 2012; 344: e1369. 22411919 10.1136/bmj.e1369

[bib13] Henry RR. Evolving concepts of type 2 diabetes management with oral medications: new approaches to an old disease. Curr Med Res Opin 2008; 24: 2189–2202. 18573228 10.1185/03007990802212981

[bib14] Kanai Y, Lee WS, You G, Brown D, Hediger MA. The human kidney low affinity Na+/glucose cotransporter SGLT2. Delineation of the major renal reabsorptive mechanism for D-glucose. J Clin Invest 1994; 93: 397–404. 8282810 10.1172/JCI116972 PMC293794

[bib15] Abdul-Ghani MA, Norton L, DeFronzo RA. Efficacy and safety of SGLT2 inhibitors in the treatment of type 2 diabetes mellitus. Curr Diab Rep 2012; 12: 230–238. 22528597 10.1007/s11892-012-0275-6

[bib16] Komoroski B, Vachharajani N, Feng Y, Li L, Kornhauser D, Dapagliflozin Pfister M. a novel, selective SGLT2 inhibitor, improved glycemic control over 2 weeks in patients with type 2 diabetes mellitus. Clin Pharmacol Ther 2009; 85: 513–519. 19129749 10.1038/clpt.2008.250

[bib17] Devineni D, Morrow L, Hompesch M, Skee D, Vandebosch A, Murphy J et al. Canagliflozin improves glycaemic control over 28 days in subjects with type 2 diabetes not optimally controlled on insulin. Diabetes Obes Metab 2012; 14: 539–545. 22226086 10.1111/j.1463-1326.2012.01558.x

[bib18] Suzuki M, Honda K, Fukazawa M, Ozawa K, Hagita H, Kawai T et al. Tofogliflozin, a potent and highly specific sodium/glucose cotransporter 2 inhibitor, improves glycemic control in diabetic rats and mice. J Pharmacol Exp Ther 2012; 341: 692–701. 22410641 10.1124/jpet.112.191593

[bib19] Nagata T, Fukazawa M, Honda K, Yata T, Kawai M, Yamane M et al. Selective SGLT2 inhibition by tofogliflozin reduces renal glucose reabsorption under hyperglycemic but not under hypo- or euglycemic conditions in rats. Am J Physiol Endocrinol Metab 2013; 304: E414–E423. 23249697 10.1152/ajpendo.00545.2012

[bib20] Kadowaki T, Ikeda S, Takano Y, Cynshi O, Christ AD, Boerlin V et al. Tofogliflozin, a novel and selective SGLT2 inhibitor improves glycemic control and lowers body weight in patients with type 2 diabetes mellitus inadequately controlled on stable metformin or diet and exercise alone. Diabetes 2012; 61(Suppl 1): A22 (abstract 80-OR).

[bib21] Ikeda S, Takano Y, Cynshi O, Christ AD, Boerlin V, Beyer U et al. A novel and selective SGLT2 inhibitor, tofogliflozin improves glycaemic control and lowers body weight in patients with type 2 diabetes mellitus. Diabetologia 2012; 55(Suppl 1): S316 (abstract 768). 10.1111/dom.12538 26179482

[bib22] Ohtake Y, Sato T, Kobayashi T, Nishimoto M, Taka N, Takano K et al. Discovery of tofogliflozin, a novel C-arylglucoside with an O-spiroketal ring system, as a highly selective sodium glucose cotransporter 2 (SGLT2) inhibitor for the treatment of type 2 diabetes. J Med Chem 2012; 55: 7828–7840. 22889351 10.1021/jm300884k

[bib23] Rodt T, Luepke M, Boehm C, von Falck C, Stamm G, Borlak J et al. Phantom and cadaver measurements of dose and dose distribution in micro-CT of the chest in mice. Acta Radiol 2011; 52: 75–80. 21498330 10.1258/ar.2010.100059

[bib24] Obata A, Kubota N, Kubota T, Sato H, Sakurai Y, Fukazawa M et al. Tofogliflozin improves insulin resistance as well as glucose tolerance by ameliorating fatty liver and obesity. Diabetes 2013; 61(Suppl 1): 1074.

[bib25] Devenny JJ, Godonis HE, Harvey SJ, Rooney S, Cullen MJ, Pelleymounter MA. Weight loss induced by chronic dapagliflozin treatment is attenuated by compensatory hyperphagia in diet-induced obese (DIO) rats. Obesity (Silver Spring) 2012; 20: 1645–1652. 22402735 10.1038/oby.2012.59

[bib26] Liang Y, Arakawa K, Ueta K, Matsushita Y, Kuriyama C, Martin T et al. Effect of canagliflozin on renal threshold for glucose, glycemia, and body weight in normal and diabetic animal models. PLoS One 2012; 7: e30555. 22355316 10.1371/journal.pone.0030555 PMC3280264

[bib27] Bolinder J, Ljunggren O, Kullberg J, Johansson L, Wilding J, Langkilde AM et al. Effects of dapagliflozin on body weight, total fat mass, and regional adipose tissue distribution in patients with type 2 diabetes mellitus with inadequate glycemic control on metformin. J Clin Endocrinol Metab 2012; 97: 1020–1031. 22238392 10.1210/jc.2011-2260

[bib28] Nagata T, Fukuzawa T, Takeda M, Fukazawa M, Mori T, Nihei T et al. Tofogliflozin, a novel sodium-glucose co-transporter 2 inhibitor, improves renal and pancreatic function in db/db mice. Br J Pharmacol 2013; 170: 519–531. 23751087 10.1111/bph.12269 PMC3791991

[bib29] Arakawa K, Ishihara T, Oku A, Nawano M, Ueta K, Kitamura K et al. Improved diabetic syndrome in C57BL/KsJ-db/db mice by oral administration of the Na^+^-glucose cotransporter inhibitor T-1095. Br J Pharmacol 2001; 132: 578–586. 11159708 10.1038/sj.bjp.0703829 PMC1572576

[bib30] Benoit SC, Clegg DJ, Seeley RJ, Woods SC. Insulin and leptin as adiposity signals. Recent Prog Horm Res 2004; 59: 267–285. 14749506 10.1210/rp.59.1.267

[bib31] Fukao T, Lopaschuk GD, Mitchell GA. Pathways and control of ketone body metabolism: on the fringe of lipid biochemistry. Prostaglandins Leukot Essent Fatty Acids 2004; 70: 243–251. 14769483 10.1016/j.plefa.2003.11.001

[bib32] Seino H, Kaku K, Maekawa S, Inagaki N, Kashiwagi A. Novel therapeutic agents for the treatment of diabetes (including SGLT2 and GPR40). J Jpn Diabetes Soc 2012; 55(Suppl 1): S–10.

[bib33] Klop B, Wouter Jukema J, Rabelink TJ, Castro Cabezas M. A physician's guide for the management of hypertriglyceridemia: the etiology of hypertriglyceridemia determines treatment strategy. Panminerva Med 2012; 54: 91–103. 22525564

[bib34] Smith BW, Adams LA. Non-alcoholic fatty liver disease. Crit Rev Clin Lab Sci 2011; 48: 97–113. 21875310 10.3109/10408363.2011.596521

[bib35] Kurosaki E, Takasu T, Yamazaki S, Koide K, Maeda N, Hastings A et al. Ipragliflozin (ASP1941), a novel SGLT2 inhibitor, demonstrates beneficial effects on nonalcoholic fatty liver disease in animal models. Diabetes 2012; 61(Suppl 1): A255 (abstract 998-P).

