# Supplementary material for: Tofogliflozin, a sodium/glucose cotransporter 2 inhibitor, attenuates body weight gain and fat accumulation in diabetic and obese animal models
Source: Nutr Diabetes. 2014 Jul 7;4(7):e125–. doi: 10.1038/nutd.2014.20 (PMC5189930; doi:10.1038/nutd.2014.20)
Supplement: Supplementary Table 1 [file nutd201420x1.pdf]

Supplementary Table 1. The lists of the reagents, animals, apparatuses and schedules for experiments

DIO rats	Animal		Male Wistar rats (Jcl), 8 weeks, n=7		
	Diet		Week -4 - -1	Week 1- 9	
		ND group	ND: D-12450B (Research Diet)	ND	
		HFD group	HFD: D-12492 (Research Diet)	HFD	
		TOFO group	HFD	HFD/TOFO (0.05%)	
	Body weight		Periodically		
	Food intake		Periodically		
	Urinary glucose		Week 1, 7		Autosera S GLU (Sekisui Medical)
	Hematocrit		Week 5, 9		Hematcrit Capillary VC-H075P (Terumo)
	Rectal temp.		Week 7, 9		Microprobe Thermomerter BAT-12 (Physitemp Instruments)
	Plasma TG		Week 9		L Type Wako TG・M (Wako)
	Plasma TC		Week 9		L Type Wako CHO・M (Wako)
	Plasma FFA		Week 9		NEFA-HA Test Wako (Wako)
	Plasma Glc		Week 9		Autosera S GLU (Sekisui Medical)
	Plasma total ketone bodies		Week 9		Auto Wako Total Ketone bodies (Wako)
	CT scan		Week 8		MicroCT Scanner eXplore Locus (General Electric Company)
	Indirect calorimetry		Week 9		O_2/CO_2 Metabolic Measuring System MM208 (Muromachi Kikai)
	Locomotor activity		Week 9		Supermex Miltichannel activity-counting System Animex Auto MK-110 Muromachi Kikai)
	Plasam insulin		Week 9		Rat insulin ELISA kit (Morinaga)
	Plasma leptin		Week 9		Rat leptin ELISA kit (Morinaga)
	Tissue weight		Week 10		
	CD14, CD68 mRNA		Week 10		7900HT Fast Real Time PCR system (Applied Biosystems)
	Size of adipocyte		Week 10		HE stainning of mesentric adipose tissue slice, NIS-Elements D2.20 SP1 (Nikon)
	Infirtration of macrophage into adipose tissue		Week 10		Immunohistochemical stainning of mesenteric adipose tissue slice with anti-rat CD68 antibody, MCA341R (AbD Serotec)<br>eSlide Capture Device Aperio ScanScope (Aperio Technologies)
KKAy mice					
Exp. 1	Animal		Male KKAy mice (Clea Japan), 8 weeks, n=12		
	Diet		Week 1 - 5		
		Control group	CE-2 (Clea Japan)		
		TOFO group	CE-2/TOFO (0.015%)		
	Body weight		Periodically		
	Food intake		Periodically		
	Plasma glucose		Day -2, 14, 28		Blood Glucose Monitoring System ACCU-Check Aviva (Roche Diagnostics)
	Glycated hemoglobin		Day -2, 28		Auto Wako HbA1c (Wako)
	Plasma TG		Day 28		L Type Wako TG・M (Wako)
	Plasma TC		Day 28		L Type Wako CHO・M (Wako)
	Plasma insulin		Day 28		Mouse insulin ELISA kit (Morinaga)
	Plasma adiponectin		Day 28		Mouse/Rat adiponectin ELISA kit (Otsuka Pharmaceutical)
	Liver weight		Day 35		
	Liver TG content		Day 35		Triglyceride E-test kit (Wako)
	Urinary glucose		Day 29 - 30		Autosera S GLU (Sekisui Medical)
Exp. 2	Animal		Male KKAy mice (Clea Japan), 8 weeks, n=8, 16		
	Diet		Day 1 - 20		
		Control group	CE-2		
		TOFO group	CE-2/TOFO (0.015%)		
	Body weight		Periodically		
	Plasam glucose		Day -1, 2, 14, 20		Blood Glucose Monitoring System ACCU-Check Aviva (Roche Diagnostics)
	Body water content		Day 3, 20		Dead body was dried at 65 ℃
Exp. 3	Animal		Male KKAy mice (Clea Japan), 8 weeks, n=6		
	Diet		Week 1- 4		
		Control group	CE-2		
		PIO group	CE-2/PIO (0.02%)		
		TOFO group	CE-2/TOFO (0.0015%)		
		PIO+TOFO group	CE-2/PIO+TOFO		
	Body weight		Periodically		
	Food intake		Periodically		
	Plasma glucose		Day -2, 14, 28		Blood Glucose Monitoring System ACCU-Check Aviva (Roche Diagnostics)
	Glycated hemoglobin		Day -2, 28		Auto Wako HbA1c (Wako)
