# Supplementary material for: Tofogliflozin, a sodium/glucose cotransporter 2 inhibitor, attenuates body weight gain and fat accumulation in diabetic and obese animal models
Source: Nutr Diabetes. 2014 Jul 7;4(7):e125–. doi: 10.1038/nutd.2014.20 (PMC5189930; doi:10.1038/nutd.2014.20)
Supplement: Supplementary Table 2 [file nutd201420x2.pdf]

Supplementary Table 2 Result of multiple regression analysis of 3 independent variables (fat mass, bone mass, lean body mass) compared to body weight gain between Week 1 and Week 8

Model's properties	Independent variables	β	t	p	Partial R^2
R^2=0.783, adj. R^2=0.745	Fat mass	0.67	7.59	7.45×10^{-7}	0.799
F=10.18, df=3	Bone mass	5.41	0.61	0.550	0.112
$p=7.01\times10^{-6}$	Lean body mass	0.18	0.59	0.564	0.074
	(constant)	-56.63	-1.44		

β is standardized regression coefficient. Partial R^2 is the partial correlation of each independent variable with body weight gain.
